# Brain matters: from environmental ethics to environmental neuroethics

**DOI:** 10.1186/s12940-016-0114-3

**Published:** 2016-02-15

**Authors:** Laura Y. Cabrera, Jordan Tesluk, Michelle Chakraborti, Ralph Matthews, Judy Illes

**Affiliations:** National Core for Neuroethics, Division of Neurology, Department of Medicine, University of British Columbia, 2211 Wesbrook Mall, Koerner S124, Vancouver, V6T 2B5 B.C. Canada; Center for Ethics & Humanities in the Life Sciences, Department of Translational Science and Molecular Medicine, Michigan State University, East Fee Hall, 965 Fee Road, Rm C211, East Lansing, MI 48823 USA; Department of Sociology, University of British Columbia, Vancouver, B.C. Canada

**Keywords:** Brain health, Mental health, Environment, Ethics, Social implications

## Abstract

The ways in which humans affect and are affected by their environments have been studied from many different perspectives over the past decades. However, it was not until the 1970s that the discussion of the ethical relationship between humankind and the environment formalized as an academic discipline with the emergence of environmental ethics. A few decades later, environmental health emerged as a discipline focused on the assessment and regulation of environmental factors that affect living beings. Our goal here is to begin a discussion specifically about the impact of modern environmental change on biomedical and social understandings of brain and mental health, and to align this with ethical considerations. We refer to this focus as Environmental Neuroethics, offer a case study to illustrate key themes and issues, and conclude by offering a five-tier framework as a starting point of analysis.

## Background: At the crossroads of environment, brain and mental health

Humans have altered their environments in pursuit of self-improvement and better opportunities since ancient times, but the scope and impact of these changes are unprecedented today [1]. Technological advancements have yielded positive economic growth, improved standards of living, and provided new ways of protecting human health. At the same time, technology has contributed to widespread negative changes in the environment that include global climate change, deforestation, suburban sprawl, ecosystem loss, and increased health risks from exposure to radiation, toxicants, and stress.

While there are different views among scholars of environmental ethics about why humans should value the environment [2], a common position focuses on direct and potential consequences to human health and well-being [3]. Environmental health experts similarly focus on environmental changes in terms of their impact on human health. However, within approaches to environmental ethics and environmental health, less attention has been paid to the specific ethical, social and legal implications of these changes for brain and mental health.^1^ To do so, requires that we probe the intersection of diverse biological, social and cultural contexts of human well-being.

Brain and mental health are determined by complex interactions between individual predispositions and behavior, social and economic processes, and the environment [4, 5]. Classic examples pointing to an association between neurological function and environmental changes include neurological deficits from exposure to mercury [6] and lead [7–9], various forms of air [10–14] and water pollution [15], pesticides, and solvents [16–20]. Moreover, cross-cultural studies of indigenous worldviews on identity, concepts of the self, and wellness have highlighted the direct and intimate connections between individuals and their environments [21, 22]. These studies remind us not only about cross-cultural differences involved in experiencing brain health and the environment, but also about different layers of vulnerability [23] brought forward by the impact of environmental change. Children [24], the elderly [25], workers who may be exposed occupationally to neurotoxicants [20] and people who live in the proximity of neurotoxicant sources [26] are more vulnerable than other sectors of the population. These unequal levels of exposure interacting with brain stage in development or decline, and differential effects from environmental risks are at the core of the environmental justice movement and, in regard to brain and mental health outcomes, are a central concern of Environmental Neuroethics.

Our goal here is to begin a discussion specifically about the impact of modern environmental change on biomedical and social understandings of brain and mental health, and to align this with ethical considerations. There are several reasons for thinking that this approach is timely. To start, brain and mental health disorders, many of which have important environmental factors, are leading contributors to disabilities and morbidity that produce critical public health, societal and economic impacts [27]. In addition, brain development, as well as its optimal function throughout the life of individuals, is particularly susceptible to the environment to which a person is exposed [24]. Considering the vulnerability of brains towards environmental exposures that are not easy to identify or to eliminate [24], we can see why brain and mental health are matters of global concern and social justice and, in particular, as the health risks related to environmental exposures are often distributed unequally. Thus, it becomes crucial to mitigate the negative impacts of environmental change while ensuring fair distribution of the positive ones. This balance represents a key aspect of the Environmental Neuroethics approach we present here.

## Fracking as a case study

Fuel sources with low greenhouse gas emissions are frequently advanced as a replacement to the rapid expansion in fossil fuel usage [28]. Technological advancements such as hydraulic fracturing (fracking) have now made extraction of these gas reserves profitable. The fracking process can impact the environment in various ways through the extraction and discharge of massive quantities of contaminated water, injection of various chemicals into the ground, and the disruption of the landscape with high densities of roads and well-heads that encroach on human settlements and wild habitats [29]. Like other literature on environmental change, contamination of the air and water supplies in the vicinity of fracking operations [17, 30] has been linked to health impacts that include asthma, respiratory complaints, gastro-intestinal effects and nosebleeds [31, 32]. Such contamination is also related to negative neurological effects. For example, McKenzie and colleagues [26] carried out a retrospective cohort study of 124,842 births between 1996 and 2009 in rural Colorado examining the associations between maternal proximity to fracking sites and birth outcomes. They found that births to mothers residing close to or surrounded by wells (>125 wells/mile) were twice as likely to have a neural tube defects compared to those with no wells within a 10-mile radius (OR = 2.0; 95 % CI: 1.0, 3.9, based on 59 cases).

**Fig. 1:**
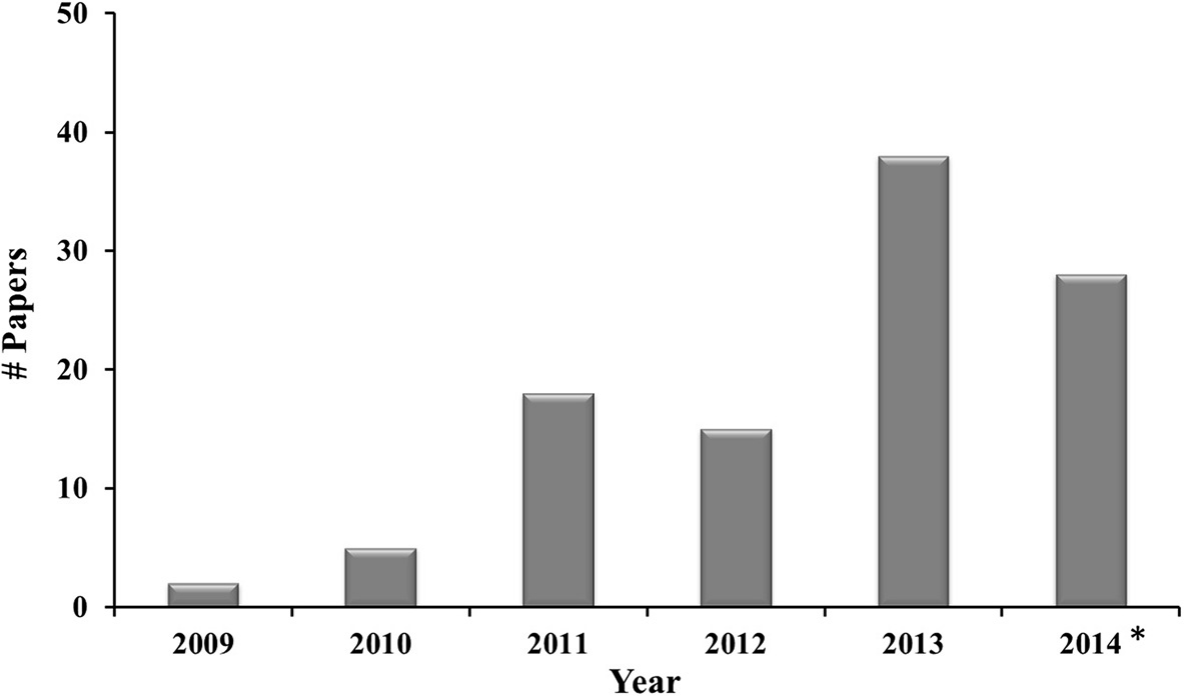
Number of articles on fracking and brain by year (*up to September 2014)

With these types of foundational studies in mind, we examined the prevalence in the literature of associations made between fracking and neurological or mental health impacts. To this end, we carried out an extensive search of peer-reviewed and gray literature of articles, theses, books, abstracts, and government reports on unconventional gas development (UGD), environment, brain and mental health using Google Scholar, the most comprehensive database relevant to the goals of the study. The searches were based on two primary key terms: (1) unconventional gas development, and (2) brain; key UGD search terms: {unconventional natural gas (+/−) development}, {shale gas (+/−) development}, {fracking} and {hydraulic fracturing}; and, key brain search terms were {brain}, {neuro}, {neurological} and {mental}. We also used a range of secondary search terms to ensure that searches identify studies relevant to culture, First Nations, health, ethics, and solastalgia.^2^ Of the one hundred and six articles identified, 83 articles originated from the peer-reviewed literature (reviews, *N* = 57; primary research *N* = 26) and 23 from the gray literature, dating back to 2009 (Fig. 1).

To provide context, we explored the origin of the cases in our sample for country of corresponding author and corresponding author disciplines. Most returns originated from the United States (USA) (*N* = 83). Twelve papers originated from Australia and six from Canada. One paper meeting our inclusion criteria originated each from China, Germany, New Zealand, Switzerland and United Kingdom. Based on the corresponding authors’ affiliation, we found that the majority of corresponding authors held multiple disciplinary associations (*N* = 45). Twenty-two held affiliations in the health sciences (e.g., medicine), 21 in the social sciences (e.g., sociology, law), 11 were associated with environmental sciences, such as ecology or forestry, and seven have disciplines represented only in a limited basis such as engineering or regional planning.

To explore the texts in depth, we conducted a three-part content analysis [33, 34] of the full set of cases. Each individual article was used as the unit of analysis. In the first phase of the analysis, we found that the dominant themes relate to public health (*N* = 31), and regulation and policy (*N* = 22). Five articles mention UGD and fracking broadly as a threat to Indigenous health.

In a second phase, we focused on brain and mental health. Eight of the 106 papers contain elaborate detailed examination of the impact that UGD poses for brain and mental health, arguments for associations between brain and mental health related to UGD, or both. The remaining papers only explore the relationship between fracking chemicals and neurotoxicity superficially and provide little if any mention of ethical implications.

In the third phase, we focused specifically on content related to ethics. Two papers provide substantial ethical discussion. One paper argues that environmental damage caused by hydraulic fracturing poses “a new threat to human rights” [35]. The other, written by members of the present author group, makes a call to the Presidential Commission for the integration of ethical considerations and neuroscience into the study of environmental change [36]. Sixty-five papers mention safety and issues related to the duty not to inflict harm; 41 papers mention at least one other ethical concern such as trust, vulnerability, justice, and disempowerment but without any further elaboration on the matter. Overall, the findings reveal that while there is emphasis on health, there is limited ethical discussion of brain and mental health impacts.

## Environmental Neuroethics in the wild

Environmental Neuroethics can provide a framework to investigate the ethical and social implications of environmental change on brain and mental health. Building on previous work [37], we propose a five-tier framework:

1. **Brain science and the environment**: Neuroscience discovery that is aligned with the measurement and evaluation of factors that affect the way individuals, communities and society adapt and cope with real or perceived environmental threats to well-being.
2. **The relational self and the environment**: The interface between the environment and brain and mental health, and the mechanisms by which exposures at key points in life may mediate different brain and mental effects; relationships among mental health stressors, susceptibility to mental health issues, and resilience within the context of changing environments.
3. **Cross-cultural factors and the environment:** Exploration of the role of culture in the relationship between environment and brain and mental health; interactions between Traditional Ecological Knowledge and neuroscience evidence; the impact of environmental change and varying effects on First Nations and settler communities given respective relationships between culture and the environment.
4. **Social policy and the environment**: Priorities and allocation of resources of local social organizations to deal with environmental impacts on brain and mental health.
5. **Public discourse and the environment:** The engagement of professional disciplines and communities in multidirectional communication and discourse about neurological, psychological, sociological and ethical dimensions of environmental change; facilitation of international, cross-disciplinary, transdisciplinary collaborations; creation of effective outreach programs that promote public understanding about the impact of environmental change on brain and mental health.

This framework can be extended more broadly to other environmental impacts such as the extraction of natural resources, air pollution, use of agricultural chemicals, water contamination, proximity to noxious facilities, mining waste and nuclear plants, ocean degradation, food contamination, and habitat destruction. Moreover, while the focus here has been on changes to the physical environment, Environmental Neuroethics is also concerned with other environments such as digital and social environments, and how these impact neurological health.

Notwithstanding the opportunity to expand ethical and social discussion around environmental change, priority setting and paths to action are not without challenges. Reliability and stability of evidence [38], knowledge of impacts [39], and appreciation of risk [40–42] are perceived and weighted differently by different stakeholders and are among the key obstacles.

## Conclusions

The identified gaps in the ethical discussion related to environmental change and health as well as the vulnerability of brains, suggest that it is time for an Environmental Neuroethics dedicated to address the interaction of biomedical and social understandings of anthropogenic environmental change. In moving forward, results and resulting scholarship and guidance must be specific, solution-oriented, and proportionate to the benefits and risks in play.

## Footnotes

1 We use the term mental health to include “well-being, everyday problems in living associated with bodily symptoms of stress and anxiety, mild depression, and seasonal fluctuations in mood and energy, as well as more severe psychiatric disorders, such as major depression, bipolar disorder, schizophrenia, and other psychotic disorders” [21 xiv].

2 A term used to refer to distress cause by environmental change.
